# The prevalence of anthropogenic nest materials differs between two distinct populations of migratory birds in Europe

**DOI:** 10.1007/s11356-023-27156-1

**Published:** 2023-05-02

**Authors:** Zuzanna Jagiello, Łukasz Dylewski, José I. Aguirre, Joanna T. Białas, Andrzej Dylik, Alejandro López-García, Ireneusz Kaługa, Adam Olszewski, Joachim Siekiera, Marcin Tobółka

**Affiliations:** 1 Department of Zoology, Poznań University of Life Sciences, Wojska Polskiego 71C, 60-625 Poznań, Poland; 2 Department of Zoology, Faculty of Sciences, University of Granada, 18071 Granada, Spain; 3 Department of Biodiversity, Ecology and Evolution, Complutense University of Madrid, José Antonio Novais, 12, 28040 Madrid, Spain; 4 Kuyavian Ornithological Region, Kotwicowa 15, 85-435 Bydgoszcz, Poland; 5 EcoLogical Group, Brzozów 19, 08-125 Suchożebry, Poland; 6 Kampinos National Park, Tetmajera 38, 05-080 Izabelin, Poland; 7 Rzeczna 17, 47-300 Żywocice, Poland; 8 Konrad Lorenz Institute of Ethology, University of Veterinary Medicine Vienna, Savoyenstraβe 1a, 1160 Vienna, Austria

**Keywords:** White stork, *Ciconia ciconia*, Nest, Plastic pollution, Birds, Human pressure, Human Footprint Index

## Abstract

A major impact of human development is the transformation of natural habitats into farming lands and the expansion of built-up areas. Also, plastic pollution is affecting wildlife on a global scale. Discarded plastic is ubiquitous and accessible for birds, which can incorporate them into the nest structure. Here, we describe the differences in type, prevalence, and the amount of anthropogenic nest materials between two populations of terrestrial, mainly farmland bird, the white stork *Ciconia ciconia*, on a broad geographical scale, from two migratory divides—eastern in Poland and western in Spain (in total 303 nests). In the two populations, we detected significant differences in the incorporation of anthropogenic nest material, as measured by the Human Footprint Index (HFI) and the Impervious Surface Areas (ISA). We found that ISA was positively related to anthropogenic nest material incorporation in the Spanish population, and HFI was positively related to anthropogenic nest material, in contrast to the Polish population, in which the relationships were not significant. Moreover, we showed that the prevalence of nests with anthropogenic nest material was two times higher in Spanish than in the Polish white stork population. This study demonstrates that the behavior of incorporation of the anthropogenic nest material differs between two distinct populations of a single bird species.

**Supplementary Information:**

The online version contains supplementary material available at 10.1007/s11356-023-27156-1.

## Introduction

Land use change is one of the most significant global threats affecting animal populations. It is observed as a transformation of natural ecosystems into farming lands, i.e., pastures and arable fields, or transformation of existing traditional agricultural lands to large-scale farming (Jeanneret et al. 2021; Raven and Wagner 2021). Together with climate change, it is rapidly increasing species extinction and ecosystem degradation (Eriksson 2021; Raven and Wagner 2021). Parallelly, the built-up areas spread worldwide and cover mainly adjacent or/and low-productive agricultural lands, particularly in developing countries (Winkler et al. 2021; Güneralp et al. 2020; Liu et al. 2020; Tilman et al. 2011). Such spread of built-up areas can be easily measured by the Impervious Surface Area (ISA), which can act as a proxy of the urbanization level in the environment (Szulkin et al. 2020). Rapid global changes in land use force wild animals to adapt to live in human-changed environments being exposed to pollution, particularly solid waste (Jagiello et al. 2022).

Among solid pollutants, plastic is the most common (Kaza et al. 2018). Global, mass-scale production of plastic started in the 1960s and 1970s (Geyer et al. 2017). It is easy to produce, versatile in use, and durable. Hence, its production is constantly increasing (Geyer et al. 2017); together with production, pollution increases. For example, in 2015, 6300 metric tonnes of plastic waste was generated worldwide, and as much as 79% (4977 metric tonnes) ended up in landfills or natural environments (Geyer et al. 2017). Unlike biodegradable materials, plastic is merely disintegrated into smaller pieces. Thus, its amount endlessly accumulates in the environment (Ter Halle et al. 2016). Therefore, plastic pollution is among the global human-induced drivers that hazard wildlife (MacLeod et al. 2021). Although it was included as the latest on the list of threats in the United Nations Environmental Programme in 2018, adverse effects, such as plastic ingestion, entrapment, and entanglement on fauna, have been known since the 1960s of the twenty-first century (Gall and Thompson 2015; Santos et al. 2021). However, studies regarding plastic pollution are considerably biased by publications on microplastic (particles of diameter < 0.5 cm) over macroplastic (particles of diameter > 2.5 cm) (Blettler et al. 2018). While a number of studies have been published recently concerning the effects of plastic pollution on terrestrial and freshwater birds, our knowledge remains biased toward studies on marine birds (Blettler and Mitchell 2021; Malizia and Monmany-Garzia 2019).

Birds are among the groups of animals studied utmost regarding land use changes and solid plastic waste pollution (Gall and Thompson 2015; Donald et al. 2001). Birds worldwide incorporate anthropogenic materials, such as plastic strings, wrapping foil, or wrapping nets in the nest (e.g., Corrales-Moya et al. 2021; Tavares et al. 2016). A recent study have shown that this behavior occurs more frequently in environments with higher human pressure (Jagiello et al., 2019). Birds may suffer adverse effects if anthropogenic materials are incorporated into nests because the materials are likely to entangle or suffocate them when ingested (Gall and Thompson 2015). Three main hypotheses can explain the incorporation of anthropogenic nest materials into avian nests: availability, age, and adaptive/functional (reviewed by Reynolds et al. 2019). According to the availability hypothesis, higher usage of anthropogenic materials is due to increased availability and accessibility in human-altered environments (Antczak et al. 2010; Lee et al. 2015). The age hypothesis proposes a relationship between the usage of anthropogenic materials and the age of the breeders (Jagiello et al. 2018; Sergio et al. 2011), while the adaptive/functional hypothesis suggests potential reproductive benefits associated with the incorporation of anthropogenic materials (Suarez-Rodriguez et al. 2012). We examined the prevalence and type of anthropogenic nest materials (ANMs) used as nesting material by the European white stork *Ciconia ciconia*. It is a large-bodied, long-lived bird species originally occurring in wetland ecosystems, but it has habituated to forage in semi-natural areas like meadows, pastures, and arable fields (Schulz 1998). Recently, this bird has been considered a farmland bird capable of serving as an indicator of biodiversity on farmland (Tobolka et al. 2012), but some populations have even become urban birds (Hmamouchi et al. 2020; Chenchouni 2017). Previously, we documented that the white storks from western and eastern migratory populations use anthropogenic nest materials (Jagiello et al. 2018, 2020). According to the results, incorporating ANM in the western population is positively related to distance to landfills and the Human Footprint Index (HFI). In the eastern population, the environmental solid waste pollution in the nest vicinity is positively related to the probability of ANM incorporation. However, previous studies quantified anthropogenic material in nests of isolated populations, and a comparative study that uses the same methodology is lacking. Our study is one of the first to examine anthropogenic materials in nests at a broader geographical scale. So far, there has only been one study demonstrating variability in ANM incorporation rates among different populations of the terrestrial species within a relatively broad geographical range (Briggs et al. 2023).

We aim to focus on a pattern of incorporation of ANM in white stork nests from two populations—western (Spain) and eastern (Poland). Firstly, we investigate whether human pressure on the environment surrounding white stork nests affects the prevalence (ratio between the number of nests with and without ANM) and the extent (the overall number of items and weight) of ANM incorporation. Secondly, we provide a detailed description of the types of ANM incorporated in white stork nests from 9 sites.

## Materials and methods

### Study sites

We collected the data in four locations in Central Spain, (1) La Torrecilla (40°18′ N, 3°37′ W), 9 nests; (2) Alcalá de Henares (40°29′ N, 3°21′ W), 8 nests; (3) Prado Herrero (40°44′ N, 3°49′ W), 25 nests; and (4) Valle del Lozoya (40°55′ N, 3°48′ W), 7 nests (data previously published in Jagiello et al. 2020), and five regions of Poland, (5) Western Poland near the town of Leszno (51°51′ N, 16°35′ E), 37 nests; (6) Southern Poland near the city of Opole (50°39′ N, 17°55′ E), 54 nests; (7) Central Poland near the town of Nakło nad Notecią (53°08′ N, 17°35′ E), 30 nests; (8) Central Poland near the city of Warsaw (52°13′ N, 21°00′ E), 54 nests; and (9) Eastern Poland near the town of Siedlce (52°10′ N, 22°16′ E), 30 nests (Figure S1). In Poland, all stork nests included in the study were solitary, while in Spain, all nests were aggregated into colonies. In Spain and North Africa, study sites varied greatly in the human presence (measured by HFI) as white storks inhabit a gradient of urban–rural habitats, nesting even in the cities forming urban populations (Hmamouchi et al. 2020; Jagiello et al. 2020). All the study sites in Poland constituted rural areas differing little in the level of human pressure (measured by HFI), as white stork nests there dominantly in agricultural landscape (Tobolka et al. 2013).

### Data collection

In both countries (Poland and Spain), we collected data during the 2018 breeding season, during ringing procedures when nestlings were between 25 and 45 days of age. Briefly, to keep the sampling homogenous, the information regarding anthropogenic nest material presence and type was only recorded for nests with successful breeding (where at least one chick was present and subsequently fledged). We did not include nests with failure or without a clutch, as they can vary significantly from successful nests regarding several breeding parameters, including nest composition (Tobolka et al. 2013). We recorded the presence or absence of anthropogenic nest materials at each visited nest. If anthropogenic nest materials were present on the surface of the nest, we collected them for detailed description and measurement. We included only anthropogenic materials from the nest surface to identify materials brought by storks in the focal breeding season. We categorized the material according to the CSIRO Global Leakage Baseline Project protocol, specifically Item List for Inland Pollution Survey (Schuyler et al., 2018; https://research.csiro.au/marinedebris/resources/), to facilitate comparisons with future studies about ANM in avian nests. We used the following categories: plastic, cloth, paper, and other (all materials other than plastic, fabric or paper; e.g., metal, glass). ANMs were weighted using an electronic scale to the nearest 1 g.

### Spatial analyses

#### Human Footprint Index (HFI)

We calculated the mean Human Footprint Index in a buffer of 2-km radius around each nest. This corresponds to the core foraging range of white storks (Zurell et al. 2018) and the range where storks collect nest material, accordingly to personal observations (Tobółka, unpublished). Due to a lack of empirical data, it was assumed that the buffer where storks collect nesting material did not differ between studied populations. Thus, the same buffer area was used for spatial analyses. The Global HFI dataset was downloaded from the NASA Socioeconomic Data and Applications Center website (http://sedac.ciesin.columbia.edu/data/set/wildareas-v2-human-footprint-geographic/data-download). This index refers to the human pressure on Earth’s surface (expressed on 1-km^2^ grid cells) and was calculated based on human population density, settlements, crop/pasture lands, roads and other access points, night-time lights, size, and remoteness of given area (Sanderson et al. 2002).

#### Impervious Surface Areas (ISA)

Similarly to HFI, in a buffer zone of a 2-km radius, mean Impervious Surface Areas were calculated for each studied nest, as Szulkin et al. (2020) described. To calculate the ISA for our dataset, we used an indicator based on satellite imagery of soil sealing/imperviousness mapping with a spatial resolution of approximately 20 m. The data was processed in 2015 by the Copernicus Land Monitoring Services and can be found at https://land.copernicus.eu/sitemap. ISA calculation includes all built-up areas, such as infrastructural networks and buildings.

Both indexes were calculated with QGIS (version 2.18.15) open access software.

### Statistical analyses

To examine differences in HFI and ISA in a 2-km buffer between populations in Poland and Spain, we used a simple Welch t-test for ISA_2000 (after log_10_ transformation) and a non-parametric U-Mann Whitney test for HFI. To determine which factors influence the presence, amount and weight of ANM, we implemented generalized linear mixed models (GLMMs) and linear mixed models (LMMs) with restricted maximum-likelihood estimator (REML). The first model (GLMM_1) included the probability of presence of ANM as a dependent variable with a binomial error structure and logit link function. The second model (GLMM_2) included the amount of ANM as a dependent variable with a negative binomial error structure. The third model (LMM_3) included ANM weight (g) (logarithm transformed: log_10_) as a dependent variable with Gaussian error structure and identity link function. In the structures of each model, we included the following predictors: white stork population (Polish and Spanish), mean Human Footprint Index in a 2-km buffer (HFI 2000), mean Impervious Surface Area in a 2-km buffer (ISA 2000) and two interactions between population and HFI 2000, and between population and ISA 2000. In all models, nest ID was used as a categorical random factor to control for the non-independence of nests, as in white stork ANM incorporation is related to the age of the builder, as older females incorporate a higher amount of anthropogenic material than younger breeders (Jagiello et al. 2018). We used Z-sore transformation to standardize explanatory variables. Multicollinearity in the explanatory variables in all models was not excessive (VIF < 2). The information-theoretic approach was employed (Burnham and Anderson 2002) to identify the most parsimonious models explaining variation in all dependent variables. Based on the full model, we constructed a set of candidate models in each analysis, calculated with maximum-likelihood (ML) estimation, that included different combinations of the predictors. We used Akaike information criterion for model selection, adjusted for small sample sizes (AICc). We used the best models with the lowest AICc values. We checked the final model validation using diagnostic plots via the DHARMa package (Hartig 2020). We carried out all the analyses in R 4.0.2 (R Core Developmental Team, 2020). GLMMs and LMM were carried out using the *lme4* (Gaussian, negative binomial and binomial distribution) package (Bates et al. 2015). The data visualizations were performed using the *ggplot2* package (Wickham, 2011).

## Results and discussion

In Spain, in a 2-km buffer around the nest, the HFI value was 61.13 ± 12.16 (mean ± SD), and the ISA value was − 6.30 ± 13.27, while in Poland, mean HFI and ISA values were 37.41 ± 8.30 and 1.48 ± 1.24, respectively. Differences were statistically significant (*U* Mann–Whitney = 126.00, *p* < 0.001 and *t* = 2.61, *p* = 0.012, respectively, for HFI and ISA).

For each study site, the values of the Human Footprint Index reflect the overall pattern of differences in human pressure between the two countries. As previously observed in Morocco and Algeria, the Spanish white stork population occupies more urbanized areas (Hmamouchi et al. 2020; Chenchouni 2017). In contrast, the Polish population remains a farmland bird and mainly occupies territories with arable lands and pastures (Tobolka et al. 2012).

In total, 34% (86 out of 254) of white stork nests contained at least one piece of incorporated anthropogenic material. However, the prevalence differed between studied populations, i.e., in Spain, 29 (59%) of 49 inspected nests contained at least one piece of anthropogenic nesting material, while in Poland, 58 (28%) of 205 inspected nests contained at least one anthropogenic item.

In terms of the amount of ANM, in Spain, 72% (21 out of 29 with ANM) of nests contained less than 10 items, while in Poland, 98% (56 out of 58 with ANM), where the majority of nests (62%) had only one item. In Spain, the overall weight of ANM items in nests smaller than 50 g was found in 55% of nests (16 out of 29 with ANM), while in Poland, 74% of nests (43 out of 58 with ANM). The weight of all anthropogenic items heavier than 100 g was present in 21% of nests in Spain (6 out of 29 with ANM) and in 7% of nests in Poland (4 out of 58 with ANM). Such a difference in prevalence may be an effect of nesting habits, as the Spanish white storks are known to occupy highly human-transformed environments and use landfills as foraging grounds and nesting material sources (López-García et al. 2021; Jagiello et al. 2020; Tortosa et al. 2003). Additionally, in Spain, white stork nests mostly colonially, unlike the studied Polish population, where birds are mostly solitary breeders. Colonial breeders rely on social information from conspecifics (Aplin 2019; Fehér et al. 2009; Hebblethwaite and Shields 1990). Therefore, individuals might copy nest-building behavior, i.e., incorporating ANM (Breen et al. 2019).

**Fig. 1:**
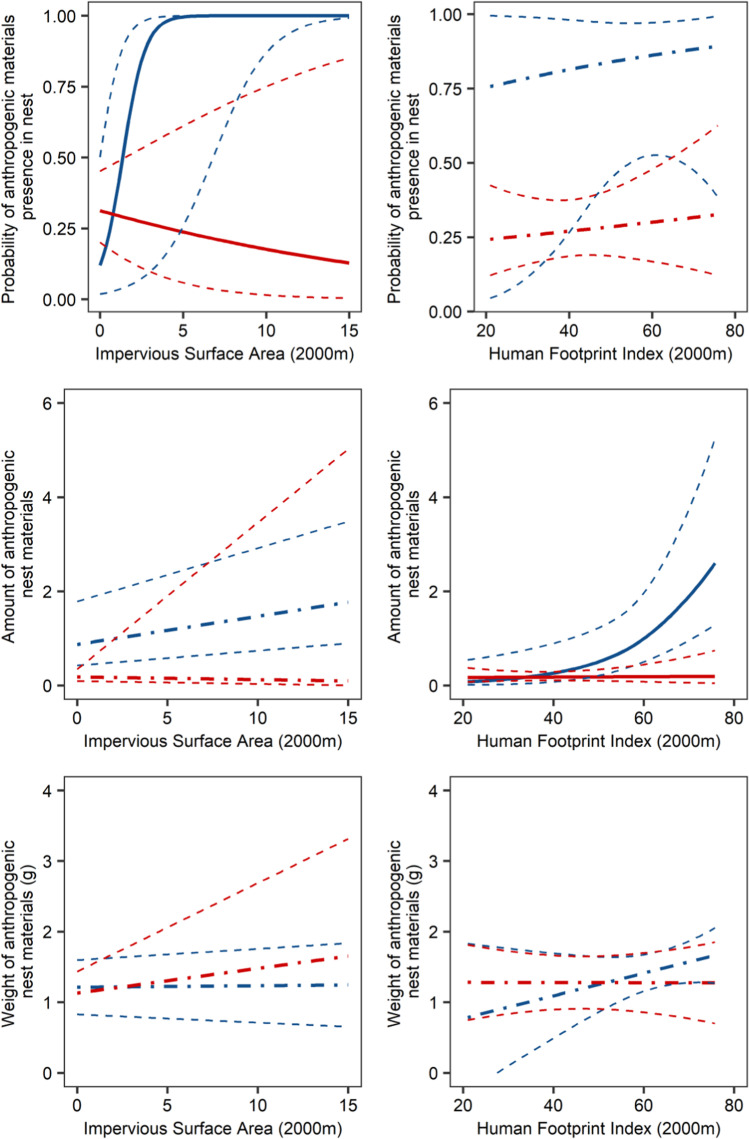
Influence of Human Footprint Index and Impervious Surface Area in 2000-m buffers around each nest, for two white stork populations (Polish population, red color, and Spanish population, blue color) on probability of anthropogenic nest materials presence (top row), amount (middle row), and weight (bottom row) of anthropogenic nest materials in white stork nests. The solid line indicates significant relationships, while dotted-point line indicates non-significant. The dotted line indicates 95% confident intervals

The probability of ANM incorporation was positively related to the urbanization level measured by ISA, whereas the amount of ANM was positively correlated with human pressure measured by HFI in the Spanish population (Table 1; Fig. 1). We did not find such relationships in Poland (Table 1; Fig. 1). Human activity measured by HFI, together with urbanization level (ISA), are proxies used for understanding the human impact on wildlife (Briggs et al. 2023; Harfoot et al. 2021; Szulkin et al. 2020; Jagiello et al., 2019). The lack of such a relationship in the Polish population is probably connected to the nesting site preferences. Storks in Poland, although known to use anthropogenic sources of food like landfills (Bialas et al. 2021) and incorporate anthropogenic nesting materials (Jagiello et al. 2018), still nest mostly in rural habitats (Tobolka et al. 2013), which are relatively homogenous in terms of human pressure. Unlike the Spanish white stork population, which nests in a gradient of natural parks and rural landscapes, it nests in human-altered environments. Due to the low variance of the predictor, there is no significant relationship between human pressure (HFI, ISA) and ANM incorporation in Poland.

**Table 1:** Model-averaged summary statistics of Generalized Linear Mixed Models (GLMMs) and linear mixed model (LMM) testing the effect of white stork population—Polish and Spanish (population), urbanization intensity in 2000 m buffer (ISA 2000), Human Footprint Index in 2000 m buffer (HFI 2000)—and two interactions: Country × ISA 2000 and Country × HFI 2000 on probability of anthropogenic nest materials presence (binomial distribution), amount of anthropogenic nest materials (Poisson distribution), and weight (g) (Gaussian distribution [after log_10_ transformation])

Variables	Estimate	SE	Z value	*p* value
Probability of anthropogenic nest materials presence				
Intercept	1.734	0.90	1.92	0.055
Population:PL	** − 2.715**	**0.96**	**2.82**	**0.005**
ISA 2000	**9.089**	**3.38**	**2.68**	**0.007**
HFI 2000	0.154	0.44	0.35	0.727
Population:PL × ISA 2000	** − 9.486**	**3.49**	**2.71**	**0.007**
Population:PL × HFI 2000	** − **0.147	0.80	0.18	0.854
Amount of anthropogenic nest materials				
Intercept	** − **2.780	2.16	1.28	0.199
Population:PL	0.802	2.32	0.34	0.730
ISA 2000	0.019	0.04	0.54	0.587
HFI 2000	0.06	0.03	1.84	0.066
Population:PL × ISA 2000	** − **0.08	0.13	0.63	0.530
Population:PL × HFI 2000	** − 0.06**	**0.03**	**2.06**	**0.039**
Anthropogenic nest materials weight (g)				
Intercept	1.358	0.14	9.87	** < 0.0001**
Population:PL	** − **0.239	0.21	1.13	0.257
ISA 2000	**0.158**	**0.08**	**1.96**	**0.050**
HFI 2000	**0.193**	**0.09**	**2.04**	**0.042**
Population:PL × ISA 2000	0.201	0.61	0.32	0.748
Population:PL × HFI 2000	** − **0.298	0.20	1.49	0.138

Nest ID was fitted as random effect. Significant results are marked in bold

Regarding the type of ANM, plastic was the most dominant material used in nests in both populations. In 89% of nests containing ANM in Spain, we found plastic, while in Poland, 67%. In both populations, the most prevalent types were plastic foil and string. We found the cloth in 45% of nests in Spain, mainly in the form of wet tissue, while in Poland, the fabric was found in 40% of nests, mainly in the form of pieces of cloth (straps, gloves, socks). We found paper in 41% of nests in Spain, dominantly as pieces of carton, while in Poland, paper was found in 14% of nests, with carton as a dominant type. In both populations, items which did not fit the listed categories were present, but “other” types played a minor role in storks nests, with items such as wire, mineral wool, Styrofoam, and insulating foam.

Our results compare ANM incorporation between two distinct populations of the same terrestrial bird species. Currently, there is only one study comparing anthropogenic nest materials incorporation in a terrestrial environment, conducted on pied flycatchers *Ficedula hypoleuca* across 17 woodland sites in the UK. Flycatchers pick the ANM selectively according to the preferred color. Therefore, they cannot be indicators of environmental solid waste pollution (Briggs et al. 2023). As opposed to the white stork, whose behavior is related to environmental solid waste pollution for the Eastern population and to human pressure from the Western population in the closest environment (Jagiello et al. 2020, 2018). It has been demonstrated that anthropogenic nest material has been a good indicator of environmental pollution in marine birds’ nests (Bond et al. 2012; Henry et al. 2011; Tavares et al. 2016). Thus, the results of our study show it is also valid for terrestrial birds, being attractive and easy to observe by amateurs under citizen science activities (Dolata 2006). For example, in a study by Blettler and Mitchell (2021), “macroplastic as nesting material” was the most dominant encounter noted by citizens among all possible interactions between macroplastic and wildlife. Nevertheless, both populations of the studied species are under pressure of human-altered environmental changes (Wuczyński et al. 2021), and storks in both populations are nesting closer to humans in recent years (Bialas et al., 2020, López-García and Aguirre 2023). Therefore, we can expect changes in the anthropogenic material incorporation in their nests. The relationship between prevalence of ANM and white stork fitness should be monitored as a potential threat to population due to the risk of entanglement in ANM.

We demonstrated that the prevalence of anthropogenic nest materials differs between populations of a single species. We have found that the inclusion of anthropogenic nest materials into avian nests is not only influenced by anthropopressure in the environment where birds nest, but also by factors such as nesting patterns and collecting materials in landfills (Bialas et al. 2021; López-García et al. 2021). Anthropogenic materials may be incorporated into nests to varying degrees depending on the bird’s breeding habits, such as whether it is a solitary or colonial breeder. Additionally, the proximity of the bird’s nesting location to landfills may also affect the amount of anthropogenic materials found in their nests. Given the current global pollution crisis, it is essential to understand this behavior locally and on a broader level. There are many environments around the world where anthropogenic materials have become prevalent. Consequently, examining how birds and other wildlife are adapting to these changes is crucial. Analyzing how birds react to human-altered environments and the challenges they face because of human activity can be gained by studying the incorporation of anthropogenic material into their nests.

## Supplementary Information

Below is the link to the electronic supplementary material.

Supplementary file1 (DOCX 1.01 MB)

## Data Availability

Data will be available on request.
