# Supplementary material for: The prevalence of anthropogenic nest materials differs between two distinct populations of migratory birds in Europe
Source: Environ Sci Pollut Res Int. 2023 May 2;30(26):69703–10. doi: 10.1007/s11356-023-27156-1 (PMC10212808; doi:10.1007/s11356-023-27156-1)
Supplement: Supplementary file 1 — Supplementary file1 (DOCX 1.01 MB) [file 11356_2023_27156_MOESM1_ESM.docx]

**Supplementary Information**

**Anthropogenic nest materials in two distinct populations of migratory bird in Europe.**

Zuzanna Jagiello^1,2^, Łukasz Dylewski^1^, José I. Aguirre^3^, Joanna T. Białas^1^, Andrzej Dylik^4^, Alejandro López-García^3^, Ireneusz Kaługa^5^, Adam Olszewski^6^, Joachim Siekiera^7^, Marcin Tobółka^1,8^

^1^Institute of Zoology, Poznań University of Life Sciences, Wojska Polskiego 71C, 60-625 Poznań, Poland

^2^Department of Zoology, Faculty of Sciences, University of Granada, 18071 Granada, Spain.

^3^Department of Biodiversity, Ecology and Evolution, Complutense University of Madrid, José Antonio Novais, 12, 28040, Madrid, Spain.

^4^Kuyavian Ornithological Region, Kotwicowa 15, 85-435 Bydgoszcz, Poland

^5^EcoLogical Group, Brzozów 19, 08-125 Suchożebry, Poland.

^6^Kampinos National Park, Tetmajera 38, 05-080 Izabelin, Poland.

^7^Rzeczna 17, 47-300 Żywocice, Poland

^8^Konrad Lorenz Institute of Ethology, University of Veterinary Medicine Vienna, Savoyenstraβe 1a, 1160 Wien, Austria

corresponding author: Zuzanna Jagiello

email: jagiello.zuzanna@gmail.com

**ORCID ID**

Zuzanna Jagiello: https://orcid.org/0000-0003-1606-2612

Łukasz Dylewski: https://orcid.org/0000-0002-1370-7625

José I. Aguirre: https://orcid.org/0000-0002-0210-890X

Joanna T. Białas: https://orcid.org/0000-0002-0683-4001

Alejandro López-García: https://orcid.org/0000-0002-1040-7651

Marcin Tobółka: <https://orcid.org/0000-0002-4989-1524>

**Figure S1** The map showing the locations of white stork nests (grey dots) in (A) Poland and in (B) Spain.

**Table S1** Subsets of the top supported GLMMs (ΔAICc <2) and LMMs (ΔAICc <2) inferring variation in probability of anthropogenic materials presence, amount and weight of anthropogenic nest materials (g) in white stork nests in Poland and Spain.

| Model subset | df | logLik | AICc | delta | weight |
| --- | --- | --- | --- | --- | --- |
| ***Probability of anthropogenic nest materials presence*** |  |  |  |  |  |
| Intercept + Country + ISA 2000 + country × ISA 2000 | 5 | -142.11 | 294.47 | 0 | 0.71 |
| Intercept + Country + ISA 2000+ HFI 2000 + country × ISA 2000 | 6 | -141.97 | 296.29 | 1.82 | 0.29 |
| ***Amount of anthropogenic nest materials*** | | | | | |
| Intercept + Country + HFI 2000 + country × HFI2000 | 5 | -330.43 | 671.1 | 0 | 0.41 |
| ***Anthropogenic nest materials weight (g)*** | | | | | |
| Intercept + HFI 2000 | 4 | -83.34 | 175.16 | 0 | 0.463 |
| Intercept + ISA 2000 | 4 | -83.86 | 176.21 | 1.05 | 0.274 |
| Intercept + Country | 4 | -83.90 | 176.28 | 1.12 | 0.264 |
